# Determinants of congenital syphilis in Fortaleza, Brazil: A retrospective case-control study

**DOI:** 10.1371/journal.pgph.0002626

**Published:** 2023-12-06

**Authors:** Melanie Etti, Antonio Silva Lima Neto, Higor S. Monteiro, Maria Alix Leite Araújo, Geziel dos Santos de Sousa, Marcia C. Castro

**Affiliations:** 1 Department of Global Health and Population, Harvard T.H. Chan School of Public Health, Boston, Massachusetts, United States of America; 2 Epidemiological Surveillance Unit, Secretaria Municipal de Saúde, Prefeitura de Fortaleza, Fortaleza, Ceará, Brazil; 3 Health Sciences Center, University of Fortaleza (UNIFOR), Fortaleza, Ceará, Brazil; 4 Department of Physics, Universidade Federal do Ceará, Fortaleza, Ceará, Brazil; 5 Postgraduate Program in Collective Health, University of Fortaleza (UNIFOR), Fortaleza, Ceará, Brazil; PLOS: Public Library of Science, UNITED STATES

## Abstract

Congenital syphilis (CS) is a significant public health problem in Brazil. Despite efforts to increase syphilis testing and treatment among pregnant women, rates of CS in the country remain high. We conducted a retrospective case-control study to identify potential associations between the mothers’ sociodemographic characteristics, clinical factors related to the current and previous pregnancies, and the occurrence of CS among newborns in Fortaleza, a populous city with one of the highest incidences of CS in Brazil. Data from newborns diagnosed with CS between 2017 and 2020 were extracted from SINAN, the national database for notifiable diseases. Data from women who had delivered an infant with CS were extracted from SINASC, the national database for registration of live births, and linked with their infant’s data. CS cases and non-CS controls were matched by year of birth at a ratio of 1:3 respectively. Potential associations were estimated using a multivariate regression model accounting for sociodemographic, obstetric, and antenatal care-related factors. Epidemiological data from 8,744 live births were included in the analysis, including 2,186 cases and 6,588 controls. The final multivariate regression model identified increased odds of delivering an infant with CS among pregnant women and girls aged below 20 years (OR 1.29), single women (OR 1.48), women who had less than 8 years of formal education (OR 2.42), women who delivered in a public hospital (OR 6.92), women who had more than 4 previous pregnancies (OR 1.60), and women who had one or more prior fetal loss (OR 1.19). The odds of delivering an infant with CS also increased as the number of antenatal visits decreased. Women who did not attend any antenatal visits had 3.94 times the odds of delivering an infant with CS compared to women who attended 7 or more visits. Our study found that increased odds of delivering an infant with CS were highly associated with factors related to socioeconomic vulnerability. These determinants not only affect the access to essential antenatal care services, but also the continuity and quality of such preventive measures. Future policies aimed at reducing the incidence of CS should not only target those pregnant women and adolescents with identifiable risk factors for testing, but also assure high quality care, treatment and follow-up for this group.

## Introduction

Congenital syphilis (CS) is a disease caused by transplacental transmission of the spirochete *Treponema pallidum* from an infected mother to the fetus during pregnancy [1]. CS is a major cause of adverse pregnancy and neonatal outcomes globally, including stillbirth and neonatal death (which may occur in up to 40% of cases), preterm delivery, low birth weight, and congenital anomalies [2]. In 2016, approximately 661,000 cases of CS were diagnosed globally, among which, 355,000 were associated with adverse birth outcomes [2].

CS is preventable, although, it relies on the timely diagnosis of syphilis in pregnancy (ideally during the first trimester) and adequate treatment with parenteral antibiotics, with the gold standard being intramuscular (IM) benzathine penicillin [3, 4]. Despite this knowledge, the incidence of CS remains high in many parts of the world, with an uneven distribution of the disease seen between and within countries [5]. While more than 90% of CS cases globally are diagnosed in developing nations [6], Brazil has one of the highest burdens of CS in the world, accounting for the majority of CS cases diagnosed in the Western Hemisphere [7]. In 2020, the Pan American Health Organization (PAHO) reported that CS cases in Brazil represented 76% of 29,149 cases reported across the Americas during the year, with a national incidence of 7.6 cases per 1000 live births [7], significantly above the target incidence of <0.5 cases per 1000 live births recommended by the World Health Organization (WHO) [8].

**Fig 1:**
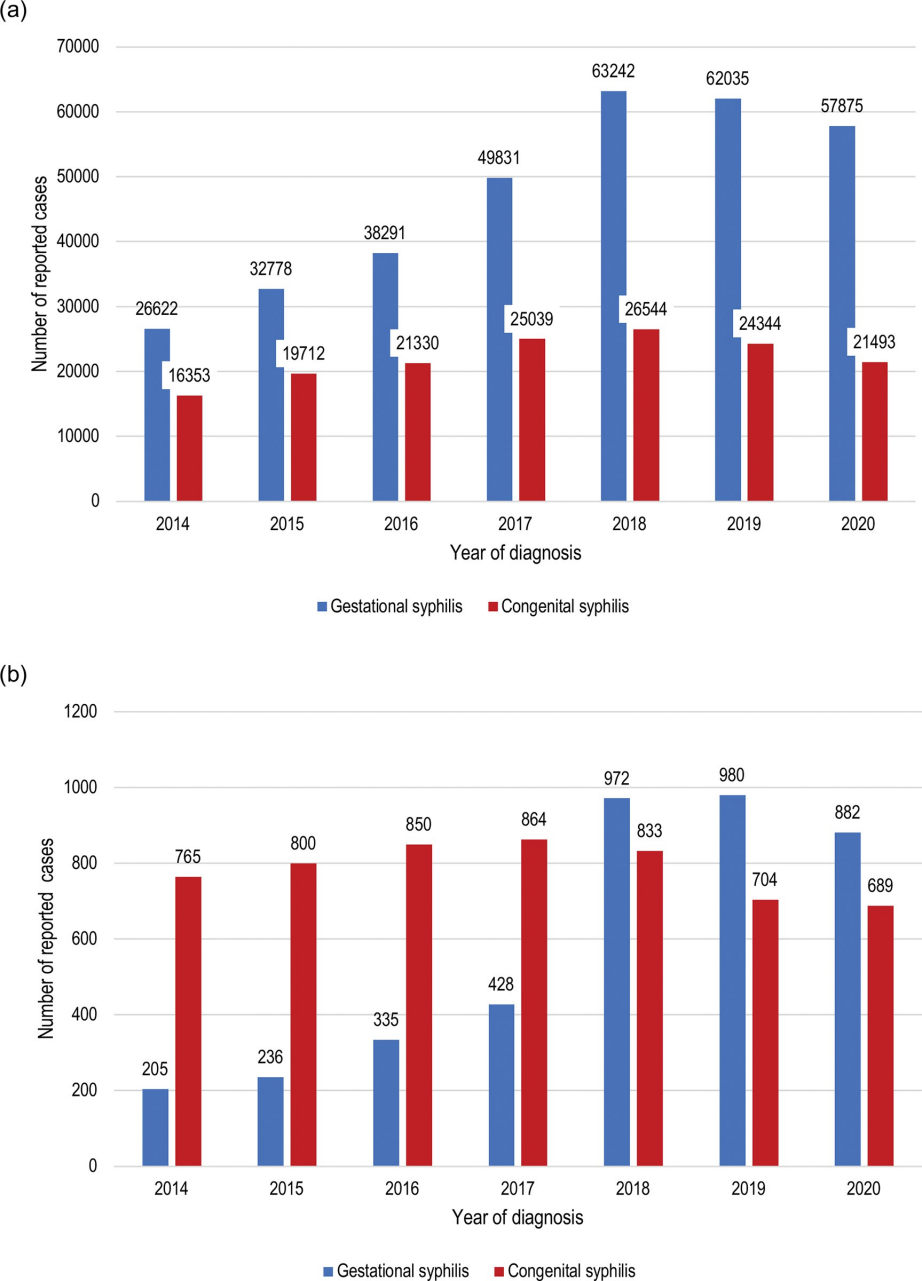
Number of reported cases of gestational and congenital syphilis between 2014–20, by year, in (a) Brazil and (b) Fortaleza (source: DATASUS) [25, 37].

In 2007, the WHO renewed efforts to eliminate CS globally by publishing a strategy for action which emphasized the importance of early antenatal care, universal antenatal syphilis screening, and prompt treatment for pregnant women diagnosed with syphilis [9, 10]. In 2010, PAHO approved a regional strategy for the dual elimination of mother-to-child transmission (EMTCT) of HIV and syphilis in Latin America and the Caribbean to address the specific challenges faced within the region [11]. This strategy aimed to strengthen surveillance systems for maternal syphilis infection and CS, build capacity within health systems for strategic and operational planning and monitoring and evaluation, and facilitate horizontal collaborations among the countries of the Americas for optimal sharing of resources and expertise [11, 12]. In the years following the introduction of this strategy, the WHO went on to validate the dual EMTCT of HIV and congenital syphilis in eight Caribbean countries between 2015 and 2021 [5]. In 2017, PAHO published the new “EMTCT Plus” framework which emphasized the need for strong political commitment to disease elimination, robust inter-programmatic planning and implementation including the integration of prevention of mother-to-child transmission (PMTCT) services into routine antenatal care, as well as high-quality surveillance of women and infants during pregnancy and after delivery through robust health information systems and improved accessibility of quality-assured diagnostic services, including point-of-care tests [13]. The updated WHO/PAHO syphilis screening in pregnancy guidelines were also published in 2017 in which the use of on-site rapid syphilis testing and the provision of the first dose of treatment for women with a positive rapid test in regions with high syphilis prevalence (5% or greater) was recommended [14]. These renewed strategies led to an increased diagnosis of gestational syphilis (GS) in Brazil from 2018 onwards (Fig 1), however, this increase was not associated with a reciprocal decline in CS cases, suggesting the persistence of the infection during pregnancy for many women with the disease [14].

**Fig 2:**
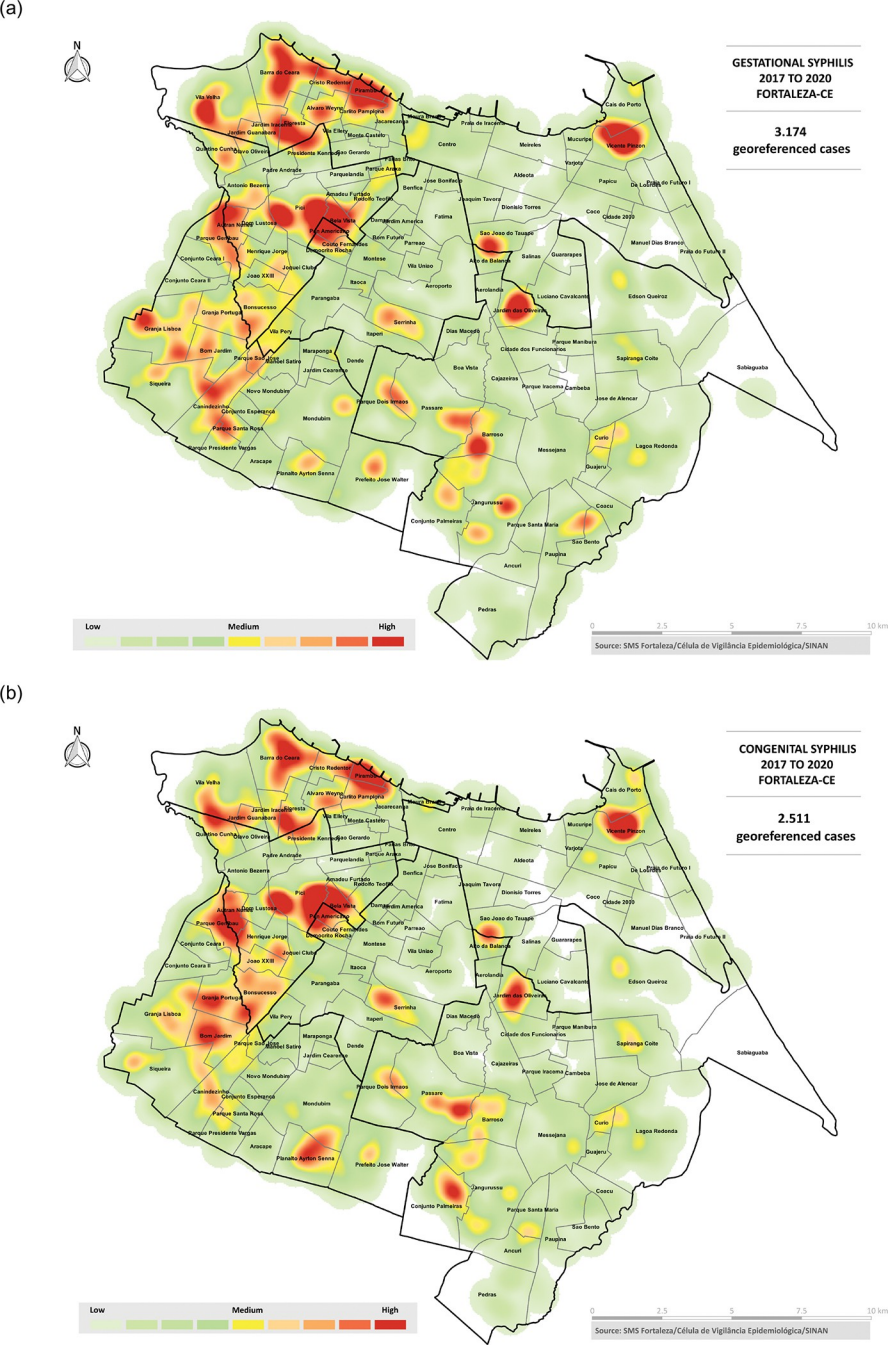
Geospatial distribution of (a) gestational and (b) congenital syphilis cases in Fortaleza between 2017–2020. Map shapefile: Brazilian Institute of Geography and Statistics (IGBE). https://www.ibge.gov.br/en/geosciences/territorial-organization/territorial-meshes/18890-municipal-mesh.html. Figure created using QGIS v3.10.

Fortaleza is the capital of the northeastern state of Ceará and the fifth most populous city in Brazil [15]. As of 2022, the city has the highest incidence of CS in Ceará and one of the highest incidences of the disease in the country [16]. Cases of CS are unevenly distributed across the city with higher disease incidence seen in some *bairros* (neighborhoods) compared to others, as shown in Fig 2. In contrast to national data trends, epidemiological surveillance data from Fortaleza revealed higher numbers of reported CS cases annually compared to reported GS cases between 2014–17, suggesting either significant under detection or underreporting of GS cases (Fig 1). While the surge in antenatal syphilis testing spurred by the updated PAHO guidance in 2017 and the updated case definition of GS (to include women with an existing diagnosis who had not completed treatment or no record of treatment completion) led to an increase in GS cases being reported in Fortaleza from 2018 onwards [17], this also was not accompanied by a notable decline in CS cases.

There have been several initiatives introduced in Brazil that have led to a decrease in the incidence of CS in many parts of the country. These efforts led to certification by the Ministry of Health of Brazil of one municipality which eliminated mother to child transmission (MTCT) of both HIV and syphilis in 2022, and 42 other municipalities that achieved either the Bronze, Silver or Gold Seal in accordance with their progress towards eliminating MTCT of these diseases [18]. In 2017 the Ministry of Health of Brazil removed completion of treatment by the mother’s sexual partner from the case definition of CS, leading to a decline in pregnant women meeting the case definition of GS in the country. One study conducted in Ceará revealed concerns among many healthcare staff in the region who felt ill-equipped to engage the sexual partners of women who have been diagnosed with syphilis during pregnancy [19], thus, it was hoped that this change would focus efforts and resources towards the identification and treatment of pregnant women with the disease to further reduce this incidence of CS. Additionally, the Syphilis No! Project was implemented in 100 municipalities in Brazil with the highest incidence of CS (of which Fortaleza was one). This project aimed to strengthen health systems to increase their capacity to diagnose and treat GS and CS, improve epidemiological surveillance systems, and develop an integrated and collaborative response to case identification [20]. An interrupted time series that evaluated the impact of the initiative between 2016 and 2019 demonstrated a greater reduction in CS cases among municipalities that were enrolled compared to those which were not, although, in some municipalities, the incidence of CS remained above the WHO target at the end of the trial period [21].

Despite these advances, further work is required for CS elimination to be achieved across Brazil. The EMTCT of syphilis remains encompassed within Sustainable Development Goal (SDG) 3.2, which aims to end all preventable deaths of newborns and children under 5 years of age globally [22]. Future efforts must build upon prior work to capture the most vulnerable members of society, including those who are at the greatest risk of this poor pregnancy outcome. It is, therefore, important that we better understand the sociodemographic, obstetric, and antenatal care-related factors associated with the delivery of an infant with CS so that pregnant women with identifiable risk factors can be targeted in future disease eradication efforts in this region. This study addresses this need. We provide a comprehensive assessment of associations between sociodemographic, obstetric, and antenatal care-related factors and the occurrence of CS among newborns in Fortaleza.

## Methods

### Study design

This retrospective case-control study was conducted using data from 2017 to 2020 extracted from two national epidemiological databases in Brazil. Healthcare professionals are mandated to report cases of CS to the Notifiable Diseases Information System (SINAN). According to the criteria adopted by the Ministry of Health of Brazil, infants with a clinical or microbiological diagnosis of syphilis within the first 12 months of life are considered cases of CS [23]. This also includes infants born to mothers diagnosed with syphilis during pregnancy who were untreated or inadequately treated for the infection [23]. The CS database within SINAN is mainly comprised of variables related to the infant’s diagnosis and details of the mother’s diagnosis of syphilis and treatment during pregnancy (if this occurred). Additional information relating to the mother, including socioeconomic status, details of any antenatal care received by the mother during the current pregnancy, and clinical information about the delivery are recorded using another form, Statement of Live Birth (DNV), which is completed by the healthcare professional attending the birth immediately after delivery and submitted electronically to the Live Births Information System (SINASC). No information relating to the infant’s diagnosis of CS and any treatment received is stored in SINASC. These two databases are distinct and do not store patient records using an identification key that could allow communication between them. To create a unified dataset linking data from the mother with that of her infant, probabilistic record linkage was performed using the Python v3.9 Record Linkage Toolkit. Controls, defined as infants without a diagnosis of CS recorded in SINAN, were randomly selected in a ratio of 3:1 to cases among newborns from SINASC, matched only by year of birth.

### Statistical analysis

Potential determinants were identified among the variables recorded in SINAN and SINASC. Following extraction of the data, the normality assumption was assessed using the Shapiro Wilk W test. Data were then described with relative frequencies calculated (Table 1). For variables with two categories, a chi-square test of homogeneity was performed and for variables with three or more categories, the likelihood-ratio chi-square test was performed to assess for differences between the two populations (Table 1). Odds ratios were calculated using logistic regression analysis. Univariate logistic regression analysis was conducted for each potential determinant and the outcome variable, CS. A multivariate model containing all significant predictors from the univariate models was constructed and pruned using backward selection. Two variables (mother’s race and month of first antenatal visit) were removed from the multivariate model as they contained missing data not at random. The final multivariate logistic regression model also controlled for regional variation in proportion of CS cases. The log-likelihood estimation was used to fit the regression models. Data analysis was performed using Stata v17.0.

**Table 1:** Descriptive table of sociodemographic characteristics, antenatal care received, and obstetric history of women who delivered infants with congenital syphilis in Fortaleza, Brazil between 2017–2020.

Variable	All n = 8744 (%)	Case n = 2186 (%)	Control n = 6558 (%)	χ^*2*^ *(p*-value)	
**MOTHER’S SOCIODEMOGRAPHIC CHARACTERISTICS**					
*Age (years)*				*143*.*67 (<0*.*001)*	
<20	1410 (16.1)	531 (24.3)	879 (13.4)		
≥20	7334 (83.9)	1655 (75.7)	5679 (86.6)		
*Marital status*				*228*.*27 (<0*.*001)*	
Single	4237 (48.5)	1365 (62.4)	2872 (43.8)		
Not single*	4341 (49.6)	783 (35.8)	3558 (54.3)		
Missing/unknown	166 (1.9)	38 (1.7)	128 (2.0)		
*Educational level*				*946*.*73 (<0*.*001)*	
<8 years	2908 (33.3)	1314 (60.1)	1594 (24.3)		
≥8 years	5560 (63.6)	825 (37.7)	4735 (72.2)		
Missing/unknown	276 (3.2)	47 (2.2)	229 (3.5)		
*Race*				*134*.*60 (<0*.*001)*	
White^&^	5149 (58.9)	1491 (68.2)	3658 (55.8)		
Not white^$^	289 (3.3)	24 (1.1)	265 (4.0)		
Missing/unknown	3306 (37.8)	671 (30.7)	2635 (40.2)		
*Human development index (HDI) of mother’s residence*				*242*.*41 (<0*.*001)*	
Low	7092 (81.1)	2007 (91.8)	5085 (77.5)		
Medium/high	690 (7.9)	92 (4.2)	598 (9.1)		
Missing/unknown	962 (11.0)	87 (4.0)	875 (13.3)		
*Region of the city where mother resides (“Regional”)*				*217*.*92 (<0*.*001)*	
Regional 1	1039 (11.9)	291 (13.3)	748 (11.4)		
Regional 2	954 (10.9)	178 (8.1)	776 (11.8)		
Regional 3	1136 (13.0)	360 (16.5)	776 (11.8)		
Regional 4	710 (8.1)	192 (8.8)	518 (7.9)		
Regional 5	1828 (20.9)	539 (24.7)	1289 (19.7)		
Regional 6	1965 (22.5)	520 (23.8)	1445 (22.0)		
Missing/unknown	1112 (12.7)	106 (4.8)	1006 (15.3)		
**ANTENATAL CARE DURING CURRENT PREGNANCY**					
*Trimester of first antenatal visit*				*134*.*06 (<0*.*001)*	
First trimester	5716 (65.4)	1132 (51.8)	4584 (69.9)		
Second/third trimester	2130 (24.3)	796 (36.4)	1334 (20.3)		
Missing/unknown	898 (10.3)	258 (11.8)	640 (9.8)		
*Number of antenatal visits*				*456*.*20 (<0*.*001)*	
None	507 (5.8)	207 (9.5)	300 (4.6)		
1–3	793 (9.1)	389 (17.8)	404 (6.2)		
4–6	2178 (24.9)	652 (29.8)	1526 (23.3)		
≥7	5169 (59.1)	880 (40.3)	4289 (65.4)		
Missing/unknown	97 (1.1)	58 (2.7)	39 (0.6)		
Type of hospital				*961*.*19 (<0*.*001)*	
Public	5874 (67.2)	2058 (94.1)	3816 (58.2%)		
Private	2492 (28.5)	80 (3.7)	2412 (36.8%)		
Missing/unknown	378 (4.3)	48 (2.2)	330 (5.0%)		
**OBSTETRIC HISTORY**					
*Number of previous pregnancies*				*250*.*43 (<0*.*001)*	
0	1628 (18.6)	397 (18.2)	1231 (18.8)		
1–3	4705 (53.8)	1215 (55.6)	3490 (53.2)		
≥4	649 (7.4)	306 (14.0)	343 (5.2)		
Missing/unknown	1762 (20.2)	268 (12.3)	1494 (22.7)		
*Number of previous fetal losses*				*82*.*02 (<0*.*001)*	
0	3726 (42.6)	996 (45.6)	2730 (41.6)		
≥1	1866 (21.3)	567 (25.9)	1299 (19.8)		
Missing/unknown	3152 (36.0)	623 (28.5)	2529 (38.6)		

* “Not single” includes individuals who reported their marital status as “married”, “widowed”, “legally separated/divorced”, or “in a stable union”

^&^ “White” includes individuals who identify as “Branca” or “Amarela”

^$^ “Not white” includes individuals who identify as “Preta”, “Parda”, or “Indígena”

### Ethical considerations

Data extraction and linkage were performed at the Fortaleza Municipal Health Secretariat, after which all data were de-identified for analysis. As the data analysis was conducted using de-identified, secondary data extracted from existing databases, this study was exempt from ethical review.

## Results

In total, 2,697 liveborn infants diagnosed with CS during the study period were identified from SINAN. Data linked from SINAN and SINASC created a unified database containing data from 2,186 liveborn infants with CS and their mothers, with a loss of 511 (18.9%) unmatched cases. The final dataset contained data from 8,744 live births: 2,186 (25%) cases and 6,558 (75%) controls. The results of the univariate and multivariate analysis are shown in Table 2.

**Table 2:** Univariate and multivariate regression analysis of association between mother’s sociodemographic characteristics, antenatal care factors, and obstetric history and congenital syphilis in Fortaleza, Brazil between 2017–2020.

Variable	Unadjusted OR (95% CI)	Adjusted OR (95% CI)
**MOTHER’S SOCIODEMOGRAPHIC CHARACTERISTICS**		
*Age (years)–Reference*: ≥*20*		
<20	2.07 (1.84–2.34)*	1.29 (1.11–1.50)*
*Marital status–Reference*: *not single*		
Single	2.13 (1.93–2.36)*	1.48 (1.32–1.66)*
*Educational level–Reference*: ≥*8 years*		
<8 years	4.69 (4.21–5.23)*	2.42 (2.15–2.72)*
*Race–Reference*: *white*		
Missing/unknown	2.71 (1.78–4.11)*	
Not white	4.34 (2.87–6.56)*	
*HDI of mother’s residence–Reference*: *medium/high*		
Missing/unknown	0.61 (0.43–0.84)*	1.60 (0.92–2.77)
Low	2.55 (2.03–3.19)*	1.25 (0.94–1.66)
**ANTENATAL CARE DURING CURRENT PREGNANCY**		
*Trimester of first antenatal visit–Reference*: *first trimester*		
Missing/unknown	1.46 (1.25–1.71)*	
Second/third trimester	1.98 (1.77–2.23)*	
*Number of antenatal visits attended–Reference*: ≥*7*		
None	3.18 (2.63–3.85)*	3.94 (3.10–5.01)*
1–3	4.44 (3.80–5.19)*	2.40 (2.02–2.85)*
4–6	1.97 (1.76–2.21)*	1.27 (1.12–1.44)*
*Type of hospital–Reference*: *private*		
Public	11.55 (9.49–14.05)*	6.92 (5.62–8.52)*
**OBSTETRIC HISTORY**		
*Number of previous pregnancies–Reference*: *0*		
Missing/unknown	0.56 (0.47–0.66)*	0.85 (0.66–1.10)
1–3	1.08 (0.09–1.23)	1.09 (0.91–1.29)
≥4	2.77 (2.28–3.35)*	1.60 (1.24–2.07)*
*Number of previous fetal losses–Reference*: *0*		
Missing/unknown	0.68 (0.60–0.77)*	1.12 (0.94–1.34)
≥1	1.19 (1.06–1.35)	1.19 (1.02–1.40)*

* Indicates *p-*value <0.05

The final multivariate regression model, which controlled for year of birth and variation in the regional incidence of CS, identified increased odds of delivering an infant with CS among pregnant women and girls aged below 20 years (OR 1.29, 95% CI 1.11–1.50), single women (OR 1.48, 95% CI 1.32–1.66), women who had less than 8 years of formal education (OR 2.42, 95% CI 2.15–2.72), women who delivered in a public hospital (OR 6.92, 95% CI 5.62–8.52), women who had more than 4 previous pregnancies (OR 1.60, 95% CI 1.24–2.07), and women who had one or more prior fetal loss (OR 1.19, 95% CI 1.02–1.40). There was a direct correlation between the odds of having an infant with CS and the number of antenatal visits attended; the odds of delivering an infant with CS increased as the number of antenatal visits decreased. Women who did not receive attend any antenatal visits during pregnancy had the highest odds (3.94, 95% CI 3.10–5.01) of delivering an infant with CS compared to women who attended 7 or more visits.

## Discussion

The results of this study identified several sociodemographic, obstetric, and antenatal care-related factors associated with the delivery of an infant with CS, many of which are associated with the socioeconomic vulnerability. Within Fortaleza, there are vast social inequalities present. While the city’s overall human development index (HDI) is 0.754 [15], this ranges from <0.2 to >0.9 across the city, with around 30% of the city’s population estimated to be living in shantytowns that span more than three-quarters of the city’s administrative districts [24]. Of interest, the odds of delivering an infant with CS were not found to be statistically different among women who lived in low HDI neighborhoods, compared to those who lived in medium or high HDI neighborhoods, suggesting that other factors may be more significant in this context.

Despite this finding, it is possible that the social inequalities across the city may be a contributing factor to the suboptimal antenatal coverage in Fortaleza. Only 66.4% of pregnant women attended seven or more antenatal visits during their pregnancy in 2020, far below the 95% recommended by the Ministry of Health of Brazil to achieve CS elimination [25, 26]. This moderate antenatal care coverage coupled with the high incidence of CS indicates an issue with both access and availability of high-quality antenatal care. CS has been described as a sentinel event reflecting poor quality antenatal care [27, 28]. Indeed, the results of this study highlight a failure in the care provided to the 880 women included in the study who attended 7 or more antenatal visits and still delivered an infant with this disease.

Of further concern, 5.8% of women included in our study did not receive any antenatal care during pregnancy, among whom the odds of delivering an infant with CS was more than three times that of the reference group (7 or more antenatal visits). These findings are in agreement with the findings of a review by Saloojee and colleagues in which it was noted that the risk of delivering an infant with CS increases when pregnant women have fewer antenatal visits or start antenatal care later in their pregnancy [29]. This finding suggests the presence of barriers to antenatal care access that may result from a number of region-specific challenges. Fortaleza has one of the highest rates of violence in the country [30], a factor linked to social deprivation which has been identified as having an adverse impact on health-seeking behaviors [31]. Additionally, the risk of intimate partner violence to women who are diagnosed with syphilis during pregnancy may also cause women to disengage with antenatal care services for fear of their safety [32].

Regarding the sociodemographic characteristics associated with delivering an infant with CS, this study identified that women and adolescents aged below 20 years of age were more likely to deliver an infant with CS than women aged 20 years or more. A cross-sectional study evaluating the maternal health of pregnant girls and women in Ceará aged between 10 to 49 years identified that pregnant adolescents were less likely to use community health services, attended fewer antenatal visits, and started antenatal care later than pregnant women who were older than 20 years of age [33]. It is possible, therefore, that there is some degree of interaction between the social, demographic, and antenatal care factors among adolescents who have delivered an infant with CS leading to this outcome.

The majority (99.2%) of women included in our study delivered their infant in a hospital, with the remaining 68 women (0.8%) delivering at home or in another clinical facility. Delivery of an infant in a public hospital was found to be associated with the greatest odds of delivering an infant with CS compared to delivery in a private hospital, although this factor is likely a proxy for other factors related to low socioeconomic status, rather than there being an increased risk of disease transmission from mother to child in public health facilities. Additionally, inconsistent reporting to the epidemiological databases from private hospitals may make CS incidence data from the private sector less reliable [34].

Several factors relating to the mother’s obstetric history were also found to be significant predictors of delivering an infant with CS. Many of these predictors are associated with lower socioeconomic status; a paper by Madalozzo *et al*. examining impact factors for transitions in fertility rates among women in Brazil found that women with lower educational attainment were more likely to have a greater number of children than women who had completed more years of education [35]. In this study, we found both factors (less than 8 years of education and four or more previous pregnancies) to be associated with increased odds of delivering an infant with CS, again highlighting a potential relationship between variables that were found to be significantly associated with increased odds of delivering an infant with CS in this study.

There were some limitations in our study. First, as previously stated, there was a loss of 18.9% of cases from our dataset. This limitation was most commonly imposed by variations in critical identifiers that limited our ability to confidently link records across the two separate epidemiological databases. Data from unmatched and matched cases were extracted from SINAN and analyzed to ensure no significant differences between the matched cases and the full dataset. It is, however, possible that our odds estimates were less precise due to the loss of data from the 511 unmatched cases. Second, data included in this study were from liveborn infants with CS and did not include stillborns. Analysis of stillborns and infants who may have died due to syphilis requires access to a different database (the Information System of Mortality, SIM) not used in this study. Third, the missingness not at random of data within the variables “race” and “trimester of first antenatal appointment” and the decision to drop these variables from the multivariate analysis may have led to omitted variable bias in our final analysis. This was ultimately deemed necessary as the inclusion of these variables would likely have reduced the precision of our multivariate model. Variables were removed in a stepwise manner to ensure their removal did not significantly affect the fit of the model. Finally, our study only included data from the first year of the COVID-19 pandemic. Preliminary epidemiological surveillance data from 2021 demonstrates a decrease in the incidence of GS cases and a rise in the incidence of CS reported in Fortaleza, likely due (at least, in part) to the limitations in healthcare access experienced by many vulnerable members of society during the pandemic. Given the adverse effect that the pandemic had on access to and provision of healthcare to women and girls in many parts of the world, including Brazil [36], we may continue to see an upsurge in CS cases in the years following this study period.

## Conclusion

In Fortaleza, there is an increasingly urgent need for targeted prevention of mother to child transmission (PMTCT) strategies that consider the varying social and healthcare needs of women and adolescents who are at the greatest risk of delivering an infant with CS. Our study supports this view by demonstrating how the socioeconomic vulnerabilities of pregnant women are highly associated with increasing odds of vertical transmission of syphilis. These determinants not only affect access to antenatal care services, but also the continuity and quality of such preventive measures. The wide differences in social circumstances means that a one-size-fits-all approach to syphilis testing and treatment may allow the most vulnerable members of the society to fall through the cracks and proceed through their pregnancy without being tested or treated for syphilis. Future initiatives could include strategies to facilitate home visitations, increased coordination between primary healthcare services and community health agents, and early warning notifications within the medical record system that can identify women and adolescents who are at risk of delivering an infant with CS. Educational programs which seek to improve health literacy among this vulnerable group may also be of benefit, and improved training of healthcare providers may increase testing and treatment of sexual partners. Ultimately, policies that not only target those who are most at risk of this adverse pregnancy outcome for testing, but also assure the high-quality treatment and follow-up in treatment will be required to reduce the incidence of CS in Fortaleza and other regions of Brazil where the burden of this disease remains unacceptably high.

## Supporting information

S1 Data (CSV)
